# Chronic Nonbacterial Osteomyelitis in Latvia from 2014 to 2025: Case Series

**DOI:** 10.3390/medicina62091642

**Published:** 2026-08-27

**Authors:** Agnese Grinēviča, Kristīne Rasa, Zane Dāvidsone

**Affiliations:** 1 Faculty of Residency, Riga Stradins University, LV-1007 Riga, Latvia; 2 Department of Education and Science, Children’s Clinical University Hospital, LV-1004 Riga, Latvia; 3 Department of Pediatric Rheumatology, Children’s Clinical University Hospital, LV-1004 Riga, Latviazane.davidsone@bkus.lv (Z.D.); 4 Department of Pediatrics, Riga Stradins University, LV-1004 Riga, Latvia

**Keywords:** chronic nonbacterial osteomyelitis (CNO), chronic recurrent multifocal osteomyelitis (CRMO), autoinflammatory bone disorder

## Abstract

*Background and Objectives*: Chronic nonbacterial osteomyelitis (CNO) is a rare pediatric autoinflammatory bone disease with variable presentation and frequent diagnostic delay. Data from Latvia are limited. This study aimed to evaluate demographic data, clinical manifestations, and treatment strategies of CNO in Latvia. *Materials and Methods*: This is a retrospective case series study of patients under 18 years of age diagnosed with chronic nonbacterial osteomyelitis (CNO) in the only pediatric tertiary hospital in Latvia between 2014 and 2025. *Results*: Thirteen patients were identified, 8 boys and 5 girls, with a median age at onset of 10 years. The pooled incidence was 0.31 per 100,000 children. The median time from symptom onset to diagnosis was 4.1 months. All patients had localized bone pain; most had fever, swelling, or a reduced range of motion. Multifocal disease occurred in all patients, with a median of 6 bones involved, and 61.5% had axial skeleton involvement. The most affected bones were the femur and tibia. Extraosseous manifestations included rash, central nervous system involvement, and lung granulomas. The most common comorbidities observed were inflammatory bowel disease and juvenile idiopathic arthritis (JIA). Inflammatory markers were moderately to markedly elevated. Whole body MRI with DWIBS was performed in all patients and showed bone marrow edema and STIR/T2 FS hyperintense lesions. All patients received NSAIDs, but only 15.4% had a complete response. Many required escalation to second-line treatment with methotrexate, TNF alpha inhibitors, or bisphosphonates. *Conclusions*: The incidence was similar to that reported in Europe. The cohort was characterized by multifocal, often severe disease with frequent fever and elevated inflammatory markers. Second-line treatment with methotrexate, TNF alpha inhibitors, or their combination showed a robust treatment response.

## 1. Introduction

Chronic nonbacterial osteomyelitis (CNO) is a noninfectious autoinflammatory disorder in children characterized by sterile, subacute, or chronic bone inflammation. Its clinical course ranges from a single bone lesion that may resolve spontaneously to aggressive, multifocal disease with recurrent flares [1,2,3]. More severe forms of CNO are referred to as chronic recurrent multifocal osteomyelitis (CRMO) [1,2]. First described by A. Giedion in 1972 [4], CNO is now considered the most common autoinflammatory bone disorder, although its true incidence remains unclear due to limited epidemiological data [3,5]. Available studies indicate that its prevalence may approach that of infectious osteomyelitis [3]. CNO typically begins in childhood, most often between 7 and 12 years of age [1,2,3,5]. In adults, a related entity, SAPHO (synovitis, acne, pustulosis, hyperostosis, and osteitis) syndrome, features sterile bone inflammation as a key component [1,6,7]. Although its pathogenesis is not fully clarified, CNO is thought to arise from dysregulation of pro-and anti-inflammatory cytokines driving persistent inflammation and osteoclast activation [1,8]. The clinical spectrum is broad, ranging from acute, localized bone pain to entirely asymptomatic lesions discovered incidentally. Early manifestations can be subtle and, combined with limited physician awareness, frequently result in delayed diagnosis [5,9]. Because its features are nonspecific, CNO remains a diagnosis of exclusion [1,9]. Whole-body MRI is helpful in detecting asymptomatic lesions and supporting the diagnosis of CNO [1,3,5]. There is currently no disease-specific therapy; treatment is empirical, targeting inflammation and bone remodeling [10].

This is the first case series reporting the pediatric experience with CNO in Latvia. Our study aims to evaluate demographic data, clinical manifestations, and treatment strategies, comparing these findings with the existing literature to enhance clinical recognition and improve diagnostic and therapeutic strategies in the future.

## 2. Materials and Methods

This retrospective case series includes pediatric patients under 18 years of age diagnosed with chronic nonbacterial osteomyelitis (CNO) in Latvia between 2014 and 2025. The diagnosis of CNO (ICD 10 code M86.3) was established by a pediatric rheumatologist, primarily based on the clinical picture and examination findings, and patient data were compared against the Jansson (2007) [11], Bristol (2016) [9], and EULAR/ACR (2025) [12] proposed criteria.

The study was conducted at the country’s only tertiary pediatric hospital, the Children’s Clinical University Hospital in Riga, ensuring national coverage. Data were collected on demographics, clinical manifestations, duration of symptoms before diagnosis, investigation results, and treatments received. Data were extracted from the information recorded in patients’ electronic medical files, including hospitalization reports and consultation follow-up reports. The annual incidence for the years 2014 to 2025 was calculated using the national health statistics database. Data were analyzed using descriptive statistical methods in Microsoft Excel version 16.106.3. Quantitative data are expressed as medians with interquartile ranges, and categorical data are presented as frequencies and percentages. Ethics Committee approval was obtained from the Research Ethics Committee of Riga Stradins University (approval number 2-4/908/2025, dated 14 August 2025).

## 3. Results

In general, 13 patients were diagnosed with chronic nonbacterial osteomyelitis between the years 2014 and 2025 at the Children’s Clinical University Hospital in Riga. Of them, 61.5% (*n* = 8) were boys and 38.5% (*n* = 5) were girls. The median age of onset was 10 years (IQR 8 to 12). The pooled incidence was 0.31 per 100,000 children (95% CI 0.15 to 0.49). The mean annual incidence is shown in Table 1.

The median time from symptom onset to diagnosis was 4.1 months (IQR 1.9 to 5.3). All patients (100%) presented with localized pain at the involved bone site. Of the 13 patients, 9 (69%) presented with local swelling, 9 (69%) had a decreased range of motion, and 10 (76.9%) presented with fever. Arthritis adjacent to the bone lesion was identified in 6 patients (46.2%). All patients had multifocal bone involvement; the median number of bones involved was 6 (IQR 5 to 8). Eight of the 13 patients (61.5%) had axial skeleton involvement. The most affected bones were the femur, tibia, fibula, pelvic bones, and mandible. In 8 of the 13 patients (61.5%), lower extremity long bone involvement was symmetrical. Bone destruction was observed in 2 patients (15.4%).

Regarding extraosseous manifestations, a rash during CNO exacerbation was observed in 1 patient (7.7%); central nervous system involvement manifesting as meningitis, cerebritis, and a brain abscess with facial nerve paresis occurred in 1 patient (7.7%); and lung granulomas were identified in 1 patient (7.7%). In 1 patient (7.7%) sensorineural hearing loss was also identified. The most common comorbidities observed in our patients were inflammatory bowel disease, which manifested concurrently with CNO in 2 patients (15.4%), and a prior diagnosis of juvenile idiopathic arthritis (JIA) in 2 patients (15.4%). Of the patients with JIA, 1 had HLA B27-positive enthesitis-related JIA and 1 had ANA-positive oligoarticular JIA with chronic uveitis. Additionally, celiac disease was present in 1 patient (7.7%).

**Figure 1:**
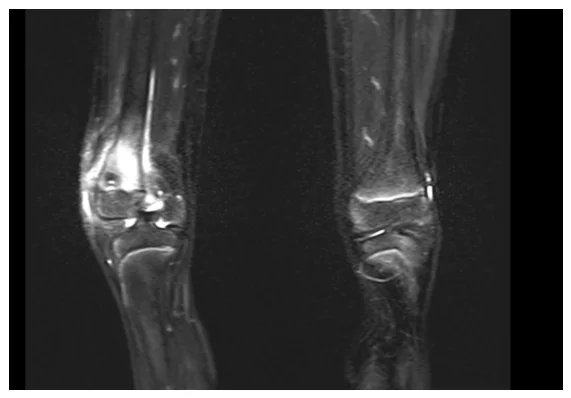
MRI imageof the right knee in a patient with CNO without bone destruction. Bone edema, a hyperintense area of 1.5 × 0.8 cm with a lower signal area around it, and a periosteal reaction in the distal metadiaphysis of the femur. Lateral and dorsal soft tissue swelling.

**Figure 2:**
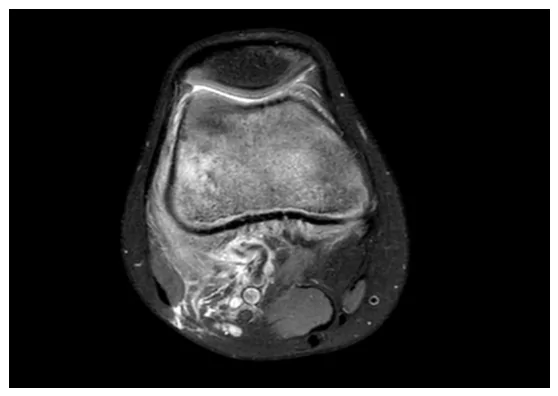
MRI image of the right knee in a patient with CNO without bone destruction. Bone edema, a hyperintense area 1.5 × 0.8 cm with a lower signal area around it, and a periosteal reaction in the distal metadiaphysis of the femur. Lateral and dorsal soft tissue swelling. An enlarged lymph node of 0.8 × 1.1 cm.

**Figure 3:**
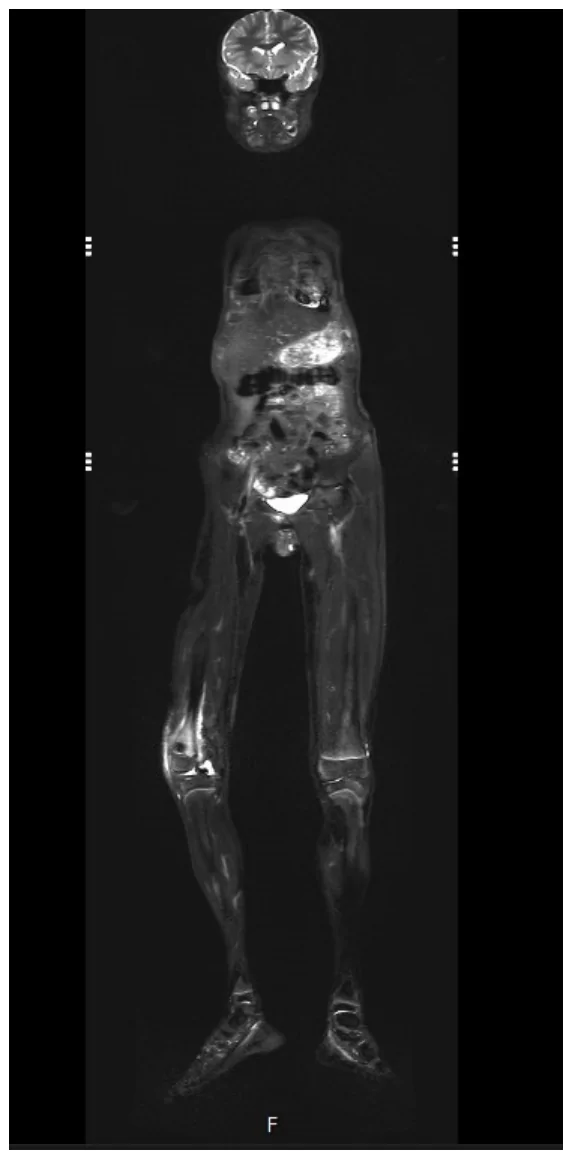
Whole-body MRI using DWIBS in a patient with CNO without bone destruction. Foci of bone marrow edema in the distal epiphysis of the right tibia and in the left femur.

**Figure 4:**
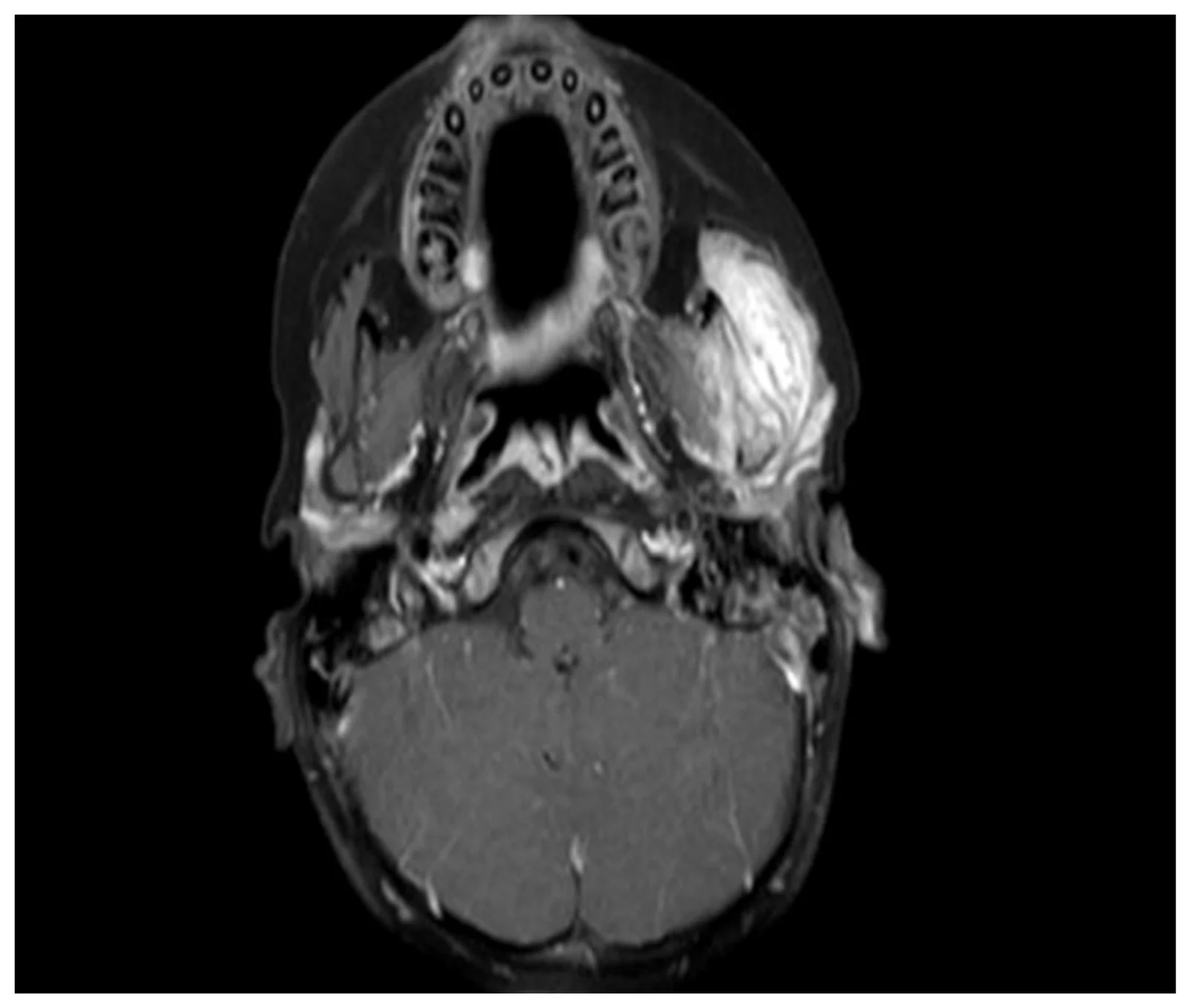
MRI of the mandible in a patient with CNO and bone destruction (detected on a computed tomography (CT) scan). Chronic inflammation in the left branch of the mandible, throughout its entire extent, extending into the body, affecting the coronoid process and angle, with perifocal soft tissue edema, extending into the masseter muscle; reactive, low-intensity inflammation in the left parotid and left submandibular salivary glands; and pronounced nonspecific lymphadenopathy.

In terms of inflammatory markers, the median CRP was 38 mg/L (IQR 5.8 to 59) and the median ESR was 55.5 mm/h (IQR 33 to 88). Genetic testing, including panels for autoinflammatory diseases, periodic fever syndromes, and primary immunodeficiencies, was done in 7 cases (53.8%). No mutations were detected. A bone biopsy was performed in 6 patients (46.2%); histological results showed nonspecific chronic inflammation in all cases, with a proportion also exhibiting fibrosis and necrotic changes. Bacterial cultures from the bone samples were negative. Whole-body MRI using DWIBS was performed in all 13 patients. The most common findings described by the radiologist on native MRI were bone marrow edema and STIR/T2 FS hyperintense lesions. Findings on region-specific MRI with contrast included patchy marrow enhancement, as well as adjacent periosteal or soft tissue enhancement or edema in some cases. MRI findings of CNO patients without bone destruction are shown in Figure 1, Figure 2 and Figure 3. An MRI image of a CNO patient with bone destruction is shown in Figure 4.

As a first-line treatment, all 13 patients received nonsteroidal anti-inflammatory drugs (NSAIDs); the most frequently prescribed were ibuprofen, naproxen, and meloxicam. Treatment with NSAIDs lasted for a duration ranging from a few days to a couple of months; only two patients (15.4%) responded to NSAIDs completely and did not require subsequent treatment with second-line agents. As a second-line treatment, six patients (46.2%) received methotrexate, and five patients (38.5%) received TNF alpha inhibitors (adalimumab, etanercept, infliximab). Four patients (30.7%) received methotrexate in combination with a TNF alpha inhibitor. Three patients (23.1%) received bisphosphonates, specifically pamidronate; one of them received monotherapy with pamidronate, and two patients received pamidronate in combination with methotrexate and a TNF alpha inhibitor. All three patients who received bisphosphonates had axial skeleton involvement. As an additional therapy to second line medications, four patients (30.8%) received glucocorticoids. Nine patients (69.2%) received antibacterial treatment prior to the CNO diagnosis. Nine of the 13 patients (69.2%) experienced only one clinically symptomatic episode of CNO; therefore, four patients (30.8%) had a recurrent course of the disease, of whom two patients (15.4%) had two clinically symptomatic episodes of CNO, one patient (7.7%) had three clinically symptomatic episodes, and one patient (7.7%) had five symptomatic episodes.

We collected data on treatment efficacy one year after establishing the diagnosis of CNO. Therapy effectiveness was evaluated based on clinical remission. Three patients (23.1%) discontinued their follow up. Of the remaining 10 patients, two (20%) were diagnosed recently, making it impossible to evaluate their therapeutic response at one year; both of these patients continue to experience symptomatic CNO episodes six months after therapy initiation. Of the eight patients with complete one-year follow-up data, five (62.5%) achieved clinical remission, while three (37.5%) continued to experience symptomatic episodes of CNO. Clinical characteristics are listed in Table 2.

**Table 2:** Clinical characteristics.

Clinical Characteristics	Number of Patients
**Symptoms at Disease Presentation**	
Localized pain	13 (100%)
Local swelling	9 (69%)
Decreased range of motion	9 (69%)
Fever	10 (76.9%)
Arthritis adjacent to the bone lesion	6 (46.2%)
**Involvement of the bones**	
Multifocal bone involvement	13 (100%)
Axial skeleton involvement	8 (61.5%)
Symmetrical long bone involvement	8 (61.5%)
Bone destruction	2 (15.4%)
**Most affected bones**	
Femur	11 (84.6%)
Tibia	11 (84.6%)
Fibula	3 (23%)
Pelvic bones	7 (53.8%)
Mandible	4 (30.7%)
**Extraosseous manifestations**	
Rash	1 (7.7%)
Central nervous system (CNS) involvement (meningitis, cerebritis, and brain abscess with facial nerve paresis)	1 (7.7%)
Sensorineural hearing loss Lung granulomas	1 (7.7%) 1 (7.7%)
**Comorbidities**	
Inflammatory bowel disease	2 (15.4%)
Juvenile idiopathic arthritis (JIA)	2 (15.4%)
Celiac disease	1 (7.7%)
**Investigation findings**	
**Genetic testing**	7 (58.3%)
No mutations were detected	
**Bone biopsy**	6 (46.2%)
Nonspecific chronic inflammation	
Proportion exhibited fibrosis	
Necrotic changes	
**MRI findings**	13 (100%)
Bone marrow edema, STIR/T2 FS hyperintense lesions (native MRI)	
Patchy marrow enhancement, adjacent periosteal or soft-tissue enhancement or edema (contrast MRI)	
**Treatment**	
NSAIDs (ibuprofen, naproxen, meloxicam)	13 (100%)
Glucocorticoids	4 (30.8%)
Methotrexate	6 (46.2%)
TNF inhibitors (adalimumab, etanercept, infliximab)	5 (38.5%)
Bisphosphonates (pamidronate)	3 (23.1%)
Antibacterial treatment prior to CNO diagnosis Glucocorticoids	9 (69.2%) 4 (30.8%)
**Number of clinically symptomatic episodes**	
1 episode of CNO	9 (69.2%)
2 episodes of CNO 3 episodes of CNO	2 (15.4%) 1 (7.7%)
5 episodes of CNO	1 (7.7%)

## 4. Discussion

This is the first study of CNO in pediatric patients in Latvia. The median age of onset was 10 years, which is consistent with other studies demonstrating that CNO onset typically occurs between 7 and 12 years [1,2,3,5]. Our incidence was 0.31 per 100,000 children, and overall, it is similar to the incidence calculated in other studies conducted in Europe. To date, only two nationwide studies were conducted in Europe: in Germany and the United Kingdom (UK) and the Republic of Ireland (ROI). The estimated incidence in the UK/ROI is 0.65 cases per 100,000 children, while the German surveillance study reported 0.4 per 100,000 children per year [5,13]. In our study, we observed a tendency of increasing incidence in the past several years that is likely due to higher clinical awareness by physicians, including pediatricians, pediatric rheumatologists, and pediatric surgeons; improved availability of MRI, especially whole-body MRI (DWIBS); and radiologists who are better trained to observe changes that raise suspicion of CNO. Increasing incidence was also observed in previously conducted studies [5]. Most studies show that girls are more commonly affected than boys, with a slight to moderate female predominance. It is also mentioned that multifocal and more severe forms of CNO can be more common in boys [1,2,13,14,15,16]. In our cohort, 61.5% of patients were boys; all the patients had multifocal bone involvement.

In our study, the median time from symptom onset to diagnosis was 4.1 months (IQR 1.9 to 5.3). There are many factors contributing to delaying diagnosis and treatment and, consequently, leading to irreversible bone damage. The most described factor that predisposes to the fact that CNO diagnosis is missed at first is nonspecific clinical symptoms mimicking other conditions, most commonly infections and malignancies. These occur alongside atypical presentation, lacking initial bone pain and presenting with systemic symptoms, like high fever or weight loss. Diagnostic challenges arising from CNO being a diagnosis of exclusion and mostly relying on imaging also contribute to late recognition. There are reports in the literature that even show a median diagnostic delay of 3.5 years [17,18,19].

Localized bone pain of usually insidious onset is the hallmark of CNO, occurring in nearly all reported patients, although pain could be absent altogether. The pain frequently worsens at night. Local swelling, tenderness over the affected bone, and warmth may also be present. Patients often present with impaired mobility, which could present as limping if the lower limb or pelvis is affected. CNO can affect any bone, but highly characteristic sites for lesions are the metaphyseal regions of the lower extremity long bones, followed by the pelvis, spine, mandible, clavicle, and long bones of the upper extremities. Involvement of the long bones may be symmetrical on both sides. Lesions near a joint can clinically manifest as arthritis. Fever usually is not common and occurs in only up to 33% of cases. Other systemic features such as malaise, fatigue, and weight loss may also be reported. The number of bone lesions varies widely among affected children, ranging from one to at least 18. In 85% of cases, multiple bones are affected with a median bone count of 3 to 4. In cases with a unifocal presentation, lesions are often multiple and detectable only through whole-body imaging. Occasionally, the disease remains asymptomatic and presents already with complications, for example, vertebral compression fractures or neurologic deficits [1,2,3]. Our study shows clinical characteristics that are consistent overall with those reported in the literature, except for the higher prevalence of fever in our patients, which was up to 76.9%.

CNO can manifest with isolated bone involvement, although it is also frequently associated with extraosseous manifestations and other inflammatory comorbidities. The most described concomitant conditions include skin manifestations, gastrointestinal comorbidities, familial Mediterranean fever (FMF), and joint involvement manifesting as ankylosing spondylitis. Skin manifestations associated with CNO are psoriasis, palmoplantar pustulosis (PPP), and acne fulminans. Gastrointestinal comorbidities are inflammatory bowel disease (IBD), including Crohn’s disease and ulcerative colitis [1,2,3,16]. Celiac disease has also been identified as a rare association in some cohorts [20]. Recent studies propose that the presence of comorbidities is significantly associated with a more severe disease phenotype, characterized by more extensive bone involvement and a greater need for intensive therapies [21,22]. In our study, two patients with CNO developed IBD, more precisely ulcerative colitis or pancolitis, and one patient with preexisting celiac disease developed CNO. We also report other extraosseous manifestations: rash during fever, meningitis, cerebritis, brain abscess, facial nerve paresis, sensorineural hearing loss, and lung granulomas. These clinical features were identified in a single patient with predominant nonbacterial osteomyelitis, substantiating the characterization of an undifferentiated autoinflammatory disorder presenting with nonbacterial osteomyelitis as a key feature.

Previous reports indicate that laboratory markers of systemic inflammation, such as C reactive protein (CRP) and erythrocyte sedimentation rate (ESR), are generally normal or only mildly to moderately elevated [1]. The frequency and degree of elevation in inflammatory markers vary significantly across different clinical studies. The previously mentioned prospective surveillance from the UK and ROI found that 86.5% of patients had a CRP level below 30 mg/L [5], but the German study noted elevated CRP in 40% of patients and elevated ESR in 72% [13]. Multicenter studies from South China [23] and the Belgian cohort [24] showed higher rates of inflammatory marker elevation. Our data show a median CRP of 38 mg/L and a median ESR of 55.5 mm/h, which are classified more appropriately as moderate to high levels of elevation of these systemic inflammatory markers, a finding that is not entirely consistent with data from previous studies. Consequently, we hypothesize that there is a possibility that at our tertiary center (Children’s Clinical University Hospital, Riga, Latvia) we recognize only more severe cases of CNO and underrecognize mild or unifocal cases of CNO. We also compared our patient data against the Jansson (2007) [11], Bristol (2016) [9], and EULAR/ACR (2025) [12] criteria. All patients met the Jansson diagnostic criteria, but not all patients met the Bristol diagnostic criteria and the EULAR/ACR classification criteria. We believe we can attribute this diagnostic discrepancy to the criteria being primarily designed for typical forms of the disease. Furthermore, according to the EULAR/ACR criteria, patients who have not undergone a bone biopsy may not accrue enough points to reach the threshold for diagnosis.

CNO is a diagnosis of exclusion (the key differential diagnoses seen in Table 3), and diagnostic confirmation relies on a combination of clinical evaluation, laboratory markers, and imaging. Whole-body MRI is the “gold standard” because it is highly sensitive and can identify asymptomatic lesions, confirming the disease’s multifocal character. DWIBS (Diffusion-Weighted Imaging with Background Body Signal Suppression) has even higher sensitivity than traditional morphological sequences alone in detecting bone marrow edema and helping distinguish benign inflammatory processes like CNO from malignant tumors [18,25]. In our study, all patients underwent whole-body MRI with DWIBS protocols. MRI findings are consistent with previous studies [1,25,26]. Bone biopsy was performed in 46.2% of patients; the data are consistent with prospective surveillance from the UK and ROI [5], but lower than in the German and Japanese studies [13,26]. With improved availability of whole-body MRI, it is possible to avoid this invasive procedure in many cases, reserving biopsy only for patients with atypical presentations [12,18,25]. Genetic testing was done in 53.8% of cases, and no mutations were detected, which is consistent with the definition of sporadic CNO. Genetic testing is not routinely required for most patients, although it is mandatory in cases where a patient’s presentation suggests a monogenic autoinflammatory syndrome (Majeed syndrome, DIRA [deficiency of IL 1 receptor antagonist], and PAPA [pyogenic arthritis, pyoderma gangrenosum, and acne] syndrome) rather than sporadic CNO [1,7].

CNO is an autoinflammatory bone disease driven by abnormal activation of the innate immune system and an imbalance between pro- and anti-inflammatory cytokines. Although its mechanisms are not fully understood, discussing the pathogenesis of CNO requires addressing several key stages, including the MAPK-ERK pathway defect, silencing of regulatory cytokines, NLRP3 inflammasome overactivation, pro-inflammatory cytokine excess, and finally, pathological bone resorption [1,2]. A selective MAPK-ERK pathway defect, while other MAPKs are activated normally, leads to silencing anti-inflammatory cytokines interleukin 10 and interleukin 19. Deficiency of interleukin 10 and interleukin 19 further leads to overactivation of the NLRP3 inflammasome, which triggers excessive release of active interleukin 1 beta and interleukin 18. In addition, overproduction of other classic pro-inflammatory cytokines, including TNF alpha, interleukin 6, and interleukin 20, occurs. This excessive production of pro-inflammatory cytokines upregulates the expression and engagement of RANK with its ligand RANKL on osteoclast precursors, further hyperactivating osteoclast differentiation and activation, leading to pathological bone resorption [1,2,10].

Treatment for CNO is primarily empirical and aimed at controlling sterile bone inflammation and preventing structural damage. There are no officially licensed medications and no evidence-based guidelines on the treatment of CNO. The choice of treatment is mostly based on personal and institutional experience, which is guided by case reports, retrospective case series, and consensus treatment plans [10,27]. As a first-line therapy, all patients received NSAIDs, a practice consistent with other studies and consensus treatment plans. However, only 15.4% of our patients responded completely to NSAIDs. This is significantly lower than the response rates in previous studies, such as the 47% to 73% response rates reported in Belgium and France [24]. As a second-line treatment based on previous studies and consensus treatment plans, the most frequently prescribed agents are classical disease-modifying anti-rheumatic drugs (DMARDs), such as methotrexate, bisphosphonates, and TNF alpha inhibitors. Regarding the pathogenesis of the disease, it is clear that bisphosphonates directly inhibit osteoclast-mediated bone resorption; additionally, bisphosphonates exert a therapeutic feedback loop by dampening the expression of pro-inflammatory cytokines in circulating monocytes. On the other hand, TNF alpha inhibitors block pathologic osteoclastogenesis as TNF alpha is a primary driver of the RANK RANKL interaction that activates osteoclasts. Classical DMARDs manage multifocal or systemic inflammation when first-line treatment fails, though they have lower relative efficacy in controlling bone-specific lesions [1,10,27]. Our data show that in more severe cases of CNO, as a second-line treatment, we chose to use methotrexate (46.2%) and TNF alpha inhibitors (38.5%) or a combination of both agents (30.7%). These findings contrast with other studies where the use of methotrexate and TNF alpha inhibitors is lower, and the most common second-line agents are bisphosphonates. A cohort from the UK and ROI showed methotrexate usage in only 3% of patients and TNF alpha inhibitor usage in 3.6% of cases. This is similar to a study from Japan where methotrexate was used in 18.5% of cases and TNF alpha inhibitors in 16.1%. The usage of bisphosphonates as second-line agents was reported to be as high as 44.8% of cases in the UK and ROI. Consequently, we hypothesize that the usage of TNF alpha inhibitors is higher in our center based on our clinical habits and greater experience working with TNF alpha inhibitors than bisphosphonates [5,26].

### Limitations

This study also has several important limitations. First, the small sample size, a cohort of just 13 patients, limits statistical power and restricts the generalizability of the findings. Second, the retrospective design and reliance on electronic medical records introduce the risk of missing, inconsistent, or variably documented data. Finally, the limited follow up and outcome data further constrain our conclusions: three patients discontinued follow up, and two lacked one-year outcome data, collectively weakening confidence in the durability and overall effectiveness of the treatment.

## 5. Conclusions

This is the first study to report clinical experience with CNO in the pediatric population in Latvia. The incidence of CNO in Latvia mirrors that reported elsewhere in Europe, and its steady rise in recent years likely reflects growing clinical awareness and improved recognition of the disease. All patients in our cohort had multifocal bone involvement, with a high median lesion count and a predominance of boys, reinforcing the notion that male patients are more likely to present with multifocal disease. The high proportion of children presenting with fever and moderately to markedly elevated inflammatory markers suggests that our cohort predominantly captures more severe CNO cases. Whole-body MRI with DWIBS proved highly valuable for identifying multifocal lesions and guiding treatment decisions. While first-line NSAID therapy induced complete remission in only a minority, many children required escalation to second-line treatment. Escalation to methotrexate, TNF alpha inhibitors, or their combination resulted in a robust treatment response.

## Figures and Tables

**Table 1:** The mean annual incidence of CNO in Latvia.

Year	No. of Patients	Children in Latvia	Incidence per 100,000 Children
2014	1	345,837	0.29
2015	0	348,660	0.00
2016	0	352,298	0.00
2017	0	356,527	0.00
2018	0	358,762	0.00
2019	3	358,813	0.84
2020	0	359,457	0.00
2021	0	358,534	0.00
2022	1	356,864	0.28
2023	2	359,030	0.56
2024	2	351,789	0.57
2025	3	343,555	1.16

**Table 3:** The key differential diagnoses of CNO to exclude (based on [1,2,18]).

Category	Disease
**Infectious diseases**	Bacterial osteomyelitis Mycobacterial infections Fungal osteitis Septic arthritis
**Malignant bone tumors and hematological disease**	Osteosarcoma Ewing sarcoma Leukemia Lymphoma Bone metastases Langerhans cell histiocytosis
**Benign bone tumors and lesions**	Osteoid osteoma Osteoblastoma Fibrous dysplasia Bone cysts Enchondromatosis Hemangiomatosis
**Inflammatory joint disorders**	Juvenile idiopathic arthritis
**Metabolic bone disorders**	Hypophosphatasia Hypertrophic osteoarthropathy
**Monogenic autoinflammatory bone diseases**	DIRA (deficiency of IL 1 receptor antagonist) Majeed syndrome PAPA (pyogenic arthritis, pyoderma gangrenosum, and acne) syndrome
**Other conditions**	Avascular osteonecrosis Osteopetrosis Hypovitaminosis C (Scurvy) Primary Immune Deficiency

## Data Availability

The data that were presented in this study are available on request from the corresponding author. The data are not publicly available, due to privacy restrictions.

## Abbreviations

The following abbreviations are used in this manuscript:

EULAR	European Alliance of Associations for Rheumatology
ACR	American College of Rheumatology
MAPK	Mitogen activated protein kinase
ERK	Extracellular signal regulated kinase
NLRP3	NLR family pyrin domain containing 3
RANK	Receptor activator of nuclear factor kappa B
RANKL	Receptor activator of nuclear factor kappa B ligand

## References

[1] Petty R.E., Laxer R., Lindsley C., Wedderburn L., Fuhlbrigge R., Mellins E. (2021). Textbook of Pediatric Rheumatology.

[2] Hedrich C.M., Hofmann S.R., Pablik J., Morbach H., Girschick H.J. (2013). Autoinflammatory bone disorders with special focus on chronic recurrent multifocal osteomyelitis (CRMO). Pediatr. Rheumatol. Online J..

[3] Hofmann S.R., Schnabel A., RŲsen-Wolff A., Morbach H., Girschick H.J., Hedrich C.M. (2016). Chronic Nonbacterial Osteomyelitis: Pathophysiological Concepts and Current Treatment Strategies. J. Rheumatol..

[4] Giedion A., Holthusen W., Masel L.F., Vischer D. (1972). Subacute and chronic “symmetrical” osteomyelitis. Ann. Radiol..

[5] Chia D.T., Toms A.P., Sanghrajka A., Ramanan A.V., Killeen O.G., Ilea C., Mahmood K., Compeyrot-Lacassagne S., Bailey K., Martin N. (2025). Incidence of chronic recurrent multifocal osteomyelitis in children and adolescents in the UK and Republic of Ireland. Rheumatology.

[6] Demirci Yildirim T., Sari I. (2024). SAPHO syndrome: Current clinical, diagnostic and treatment approaches. Rheumatol. Int..

[7] Girschick H., Finetti M., Orlando F., Schalm S., Insalaco A., Ganser G., Nielsen S., Herlin T., Koné-Paut I., Martino S. (2018). The multifaceted presentation of chronic recurrent multifocal osteomyelitis: A series of 486 cases from the Eurofever international registry. Rheumatology.

[8] Cox A.J., Zhao Y., Ferguson P.J. (2017). Chronic Recurrent Multifocal Osteomyelitis and Related Diseases—Update on Pathogenesis. Curr. Rheumatol. Rep..

[9] Roderick M.R., Shah R., Rogers V., Finn A., Ramanan A.V. (2016). Chronic recurrent multifocal osteomyelitis (CRMO)—Advancing the diagnosis. Pediatr. Rheumatol. Online J..

[10] Roberts E., Charras A., Hahn G., Hedrich C.M. (2024). An improved understanding of pediatric chronic nonbacterial osteomyelitis pathophysiology informs current and future treatment. J. Bone Min. Res..

[11] Jansson A., Renner E.D., Ramser J., Mayer A., Haban M., Meindl A., Grote V., Diebold J., Jansson V., Schneider K. (2007). Classification of non-bacterial osteitis: Retrospective study of clinical, immunological and genetic aspects in 89 patients. Rheumatology.

[12] Zhao Y., Oliver M.S., Schnabel A., Wu E.Y., Wang Z., Marino A., Aguiar C.L., Akikusa J.D., Akca U.K., Almeida B. (2026). EULAR/American College of Rheumatology Classification Criteria for Pediatric Chronic Nonbacterial Osteomyelitis. Arthritis Rheumatol..

[13] Jansson A.F., Grote V., Group E.S. (2011). Nonbacterial osteitis in children: Data of a German Incidence Surveillance Study. Acta Paediatr..

[14] Wipff J., Costantino F., Lemelle I., Pajot C., Duquesne A., Lorrot M., Faye A., Bader-Meunier B., Brochard K., Despert V. (2015). A large national cohort of French patients with chronic recurrent multifocal osteitis. Arthritis Rheumatol..

[15] Alahmadi L., Almasoud J., Almojali A., Alsuwairi W., Alrasheed A., Al-Mayouf S., Alkhars F., Alenazi A., Alenazi S., Asiri A. (2025). Chronic Non-Bacterial Osteomyelitis in Pediatric Patients: Findings from Multiple Centers in Saudi Arabia. J. Epidemiol. Glob. Health.

[16] Reiser C., Klotsche J., Hospach A., Berendes R., Schnabel A., Jansson A.F., Hufnagel M., Grösch N., Niewerth M., Minden K. (2021). First-year follow-up of children with chronic nonbacterial osteomyelitis-an analysis of the German National Pediatric Rheumatologic Database from 2009 to 2018. Arthritis Res. Ther..

[17] Karam M., Bamashmous E., Gourishankar A., DuBose J.M.W. (2025). When fever comes first: An unusual presentation of chronic non-bacterial osteomyelitis. BMJ Case Rep..

[18] Schaal M.C., Gendler L., Ammann B., Eberhardt N., Janda A., Morbach H., Darge K., Girschick H., Beer M. (2021). Imaging in non-bacterial osteomyelitis in children and adolescents: Diagnosis, differential diagnosis and follow-up-an educational review based on a literature survey and own clinical experiences. Insights Imaging.

[19] Gupta V., Jain A., Aggarwal A. (2018). Chronic nonbacterial osteomyelitis from a tertiary care referral center. J. Postgrad. Med..

[20] Costi S., Germinario S., Pandolfi M., Pellico M.R., Amati A., Gattinara M., Chighizola C.B., Caporali R., Marino A. (2023). Chronic Nonbacterial Osteomyelitis and Inflammatory Bowel Disease: A Literature Review-Based Cohort. Children.

[21] Öksüz Aydın B., Aydın F., Taş Ö., Bahçeci O., Özçakar Z.B. (2025). Associated diseases and their effects on disease course in patients with chronic non-bacterial osteomyelitis: Retrospective experience from a single center. Clin. Rheumatol..

[22] Cebecauerová D., Malcová H., Koukolská V., Kvíčalová Z., Souček O., Wagenknecht L., Bronský J., Šumník Z., Kynčl M., Cebecauer M. (2022). Two phenotypes of chronic recurrent multifocal osteomyelitis with different patterns of bone involvement. Pediatr. Rheumatol. Online J..

[23] Ma L., Liu H., Tang H., Zhang Z., Zou L., Yu H., Sun L., Li X., Tang X., Lu M. (2022). Clinical characteristics and outcomes of chronic nonbacterial osteomyelitis in children: A multicenter case series. Pediatr. Rheumatol. Online J..

[24] Kaut S., Van den Wyngaert I., Christiaens D., Wouters C., Noppe N., Herregods N., Dehoorne J., De Somer L. (2022). Chronic nonbacterial osteomyelitis in children: A multicentre Belgian cohort of 30 children. Pediatr. Rheumatol. Online J..

[25] Nico M.A.C., Araķjo F.F., Guimar„es J.B., da Cruz I.A.N., Silva F.D., Carneiro B.C., Filho A.G.O. (2022). Chronic nonbacterial osteomyelitis: The role of whole-body MRI. Insights Imaging.

[26] Maeda Y., Hiejima E., Izawa K., Nishimura K., Yazawa Y., Iwata N., Michikura M., Ishikawa H., Ito S., Nakamura Y. (2025). The first nationwide epidemiological survey of chronic recurrent multifocal osteomyelitis in Japan. Mod. Rheumatol..

[27] Zhao Y., Wu E.Y., Oliver M.S., Cooper A.M., Basiaga M.L., Vora S.S., Lee T.C., Fox E., Amarilyo G., Stern S.M. (2018). Consensus Treatment Plans for Chronic Nonbacterial Osteomyelitis Refractory to Nonsteroidal Antiinflammatory Drugs and/or With Active Spinal Lesions. Arthritis Care Res..

